# Peripheral Blood Markers Correlate with the Progression of Active Tuberculosis Relative to Latent Control of *Mycobacterium tuberculosis* Infection in Macaques

**DOI:** 10.3390/pathogens11050544

**Published:** 2022-05-05

**Authors:** Maya Gough, Dhiraj K. Singh, Chivonne Moodley, Tianhua Niu, Nadia A. Golden, Deepak Kaushal, Smriti Mehra

**Affiliations:** 1 Southwest National Primate Research Center, Texas Biomedical Research Institute, San Antonio, TX 78227, USA; mgough@txbiomed.org (M.G.); dsingh@txbiomed.org (D.K.S.); cmoodley@tulane.edu (C.M.); 2 Tulane University Health Science Center, New Orleans, LA 70112, USA; 3 Department of Biostatistics and Bioinformatics, School of Public Health and Tropical Medicine, Tulane University, New Orleans, LA 70112, USA; tniu@tulane.edu; 4 Tulane National Primate Research Center, Covington, LA 70433, USA; nabraham@tulane.edu

**Keywords:** *Mycobacterium tuberculosis*, active tuberculosis disease, latent tuberculosis infection, macaque model

## Abstract

Despite a century of research into tuberculosis (TB), there is a dearth of reproducible, easily quantifiable, biomarkers that can predict disease onset and differentiate between host disease states. Due to the challenges associated with human sampling, nonhuman primates (NHPs) are utilized for recapitulating the closest possible modelling of human TB. To establish a predictive peripheral biomarker profile based on a larger cohort of rhesus macaques (RM), we analyzed results pertaining to peripheral blood serum chemistry and cell counts from RMs that were experimentally exposed to *Mtb* in our prior studies and characterized as having either developed active TB (ATB) disease or latent TB infection (LTBI). We compared lung CFU burdens and quantitative pathologies with a number of measurables in the peripheral blood. Based on our results, the investigations were then extended to the study of specific molecules and cells in the lung compartments of a subset of these animals and their immune responses. In addition to the elevated serum C-reactive protein (CRP) levels, frequently used to discern the level of *Mtb* infection in model systems, reduced serum albumin-to-globulin (A/G) ratios were also predictive of active TB disease. Furthermore, higher peripheral myeloid cell levels, particularly those of neutrophils, kynurenine-to-tryptophan ratio, an indicator of induced expression of the immunosuppressive molecule indoleamine dioxygenase, and an influx of myeloid cell populations could also efficiently discriminate between ATB and LTBI in experimentally infected macaques. These quantifiable correlates of disease were then used in conjunction with a regression-based analysis to predict bacterial load. Our results suggest a potential biomarker profile of TB disease in rhesus macaques, that could inform future NHP–TB research. Our results thus suggest that specific biomarkers may be developed from the myeloid subset of peripheral blood or plasma with the ability to discriminate between active and latent *Mtb* infection.

## 1. Introduction

Tuberculosis (TB), caused by *Mycobacterium tuberculosis* (*Mtb*), is one of the deadliest infectious diseases facing humanity, leading to ~10 million new cases and ~1.6 million deaths annually, as per a recent report from 2021 [1]. Studies that have attempted to devise efficient, cost effective, accurate, non-invasive, forms of diagnosis experience their own set of drawbacks. In part, this is due to the fact that *Mtb* infection can have a spectrum of different outcomes inside a human host, ranging from a lifelong asymptomatic infection, termed latent tuberculosis infection (LTBI), to a reactivation of LTBI. TB can present as slowly progressing chronic pulmonary TB, rapidly fulminant pulmonary TB, as well as extra-pulmonary TB [2]. In each of these instances, the level of bacterial burden and the extent of the granulomatous pathology can differ significantly. Due to the variability of infection and host response, accurately diagnosing TB through the use of a single method is an ambitious undertaking. It is particularly difficult to identify TB positive patients in high burden areas where the specificity of routine tests is undermined by extensive BCG vaccination and the high burden of environmental mycobacteria. Despite advances in TB diagnostics, a definitive set of biomarkers for diagnosis of disease and monitoring of disease progression has not yet been identified [3]. 

Rhesus and cynomolgus macaques have proven excellent models of human *Mtb* infection [4,5]. Experimental infection of macaques can result in a plurality of outcomes [6], and the lungs of these animals exhibit a wide variety of pathological lesions reminiscent of human TB [5]. We have therefore employed the macaque model of TB to study various aspects of the host immune function [7,8,9,10,11,12,13,14,15,16,17,18,19,20], as well as the role of the bacterial virulence factors [6,19,21,22]. We have collected clinical data from rhesus macaques which progressed to active pulmonary TB disease, or which exhibited immune control over infection, which was characterized by lower bacillary burdens, indicative of LTBI.

The macaque model allows longitudinal collection of both lung and peripheral samples as well as the associated data on a repeated basis, from animals with precise infection. Thus, we can collect accurate lung tissue CFUs and determine lung pathology with a degree of precision, that is impossible with infected humans due to a variety of reasons. It is therefore critical to identify surrogate markers in the blood, which correlate with active TB, rather than infection (or BCG vaccination). Ideally, such markers would discriminate between the onset of active TB (ATB), characterized by high lung burdens and pathology, and LTBI. It may be possible to retrospectively analyze samples from a large cohort of animals and identify correlates of risk of acquiring active TB. This current study represents a preliminary step in this direction. We have collected clinical data from different cohorts of animals, which did or did not exhibit clinical TB due to immune control of infection. 

In our current analysis, we examine if complete blood cell counts, serum chemistry markers, cell populations, and tryptophan metabolism correlate with ATB versus LTBI. The levels of serum C-reactive protein (CRP) are known to correlate highly with pulmonary TB in macaques. Based on accumulating data in the field, we hypothesized that other peripheral blood-based markers, including both the accumulation of myeloid cells, as well as the myeloid cell expression of specific protein markers of immune dysfunction and/or inflammation, would also differentiate the onset of ATB versus LTBI. CRP is a marker for systemic inflammation that is produced by several cell types, most notably macrophages, lymphocytes, and endothelial cells. CRP is involved in several inflammatory pathways, including nitric oxide production and cytokine secretion [23]. The use of CRP to detect active TB was proposed previously, although several other inflammatory disorders were found to confound results in humans [24,25]. Here, we show data that the use of serum CRP levels can be coupled with other blood chemistry parameters, such as serum albumin-to-globulin (A/G) ratios and cell counts to significantly discriminate between *Mtb* infection and the onset of disease. We also found distinct differences between cell populations and tryptophan availability between ATB and LTBI primates. These results build on several observations in previous studies with humans, as well as animal models, suggesting that the timely advent of T cell recruitment correlates with control of infection, while uncontrolled infection is characterized by greater myeloid cell influx to the site of infection. 

## 2. Results

### 2.1. Establishing Lung Mtb Burdens and Pathology for ATB and LTBI in NHPs

**Figure 1:**
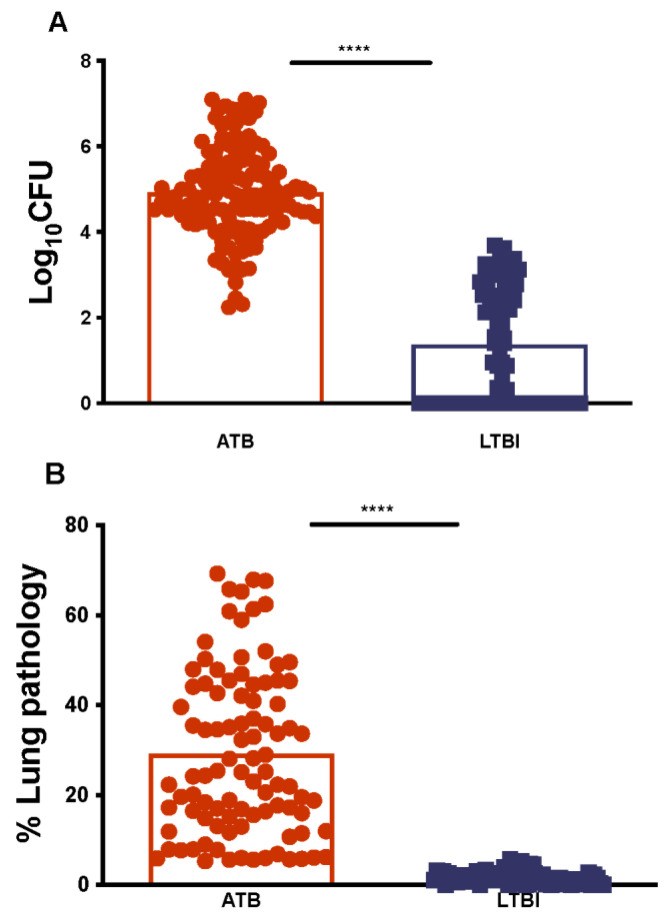
Bacterial Burdens (Log_10_CFU) and Percent Lung Pathology. Bacterial burdens (**A**) were determined from animals described in the study protocol that had been designated to either have active TB disease based on clinical attributes (red) or LTBI/control of infection (blue). (****, *p*-value of < 0.0001). (**B**) The percentage of lung pathology was computed using the previously described morphometric techniques in the animals described in the study protocol that had been designated to either have active TB disease based on clinical attributes (red) or LTBI/control of infection (blue). (****, *p*-value of < 0.0001). T-test was used to identify statistically significant differences.

Previous studies have found that lung *Mtb* burden (determined by averaging the number of bacilli recovered from a large number of randomly collected samples off a grid during necropsy) and the extent of quantifiable lung pathology (scored by morphometrics also on random samples derived at necropsy), best define the outcome of experimental infections in susceptible NHP hosts [7,12,15,17,18,19,22]. We have accumulated data from macaques with either active (ATB, n = 140) or latent (LTBI, n = 85) TB infections. These NHPs were infected with *Mtb* within the seven-year span of 2007–2014, and endpoint lung bacterial burdens and pathology scores were assessed. The lung bacterial burden for animals with ATB ranged from 3.1 × 10^3^–1 × 10^7^ CFU/gm of tissue, with 4 × 10^5^ CFU/gm being the mean bacterial burden in the lung (Figure 1A). NHPs that exhibited either LTBI or chronic asymptomatic TB, as characterized by clinical measures, had significantly lower lung burdens (Figure 1A). The burden in LTBI NHPs ranged from 1 × 10^0^–1 × 10^3^CFU/gm with a mean value of < 1 × 10^2^ CFU/gm (Figure 1A). The difference in CFU counts were highly statistically significant when comparing ATB with LTBI (*p* = 4.484 × 10^−6^, Table 1) (Figure 1A). The percentage of lung area involved in pathology amongst ATB NHPs typically ranged from 5.3–69.3% with a mean of 29.2% (Figure 1B), whereas LTBI NHPs ranged from 0–5.8% with a mean of 1.8%. These differences were highly statistically significant when adjudged using a Welch’s t test (*p* < 0.0001, Figure 1B). Our results show that lung burden and pathology distinguish the two main outcomes of pulmonary *Mtb* infection; however, these two measures of disease require extensive sampling of the lung tissue that is generally only possible post-mortem.

### 2.2. Serum CRP Levels Compared between ATB and LTBI

We observed that the elevation of serum CRP levels and serum A/G ratios in macaques with *Mtb* infection corresponded with either lung bacterial burdens or pulmonary pathology. Serum CRP levels serve as excellent markers of inflammation. In previous studies with both rhesus and cynomolgus macaques experimentally infected with *Mtb*, CRP has served as a specific measure to identify correlates of TB disease progression [6,7,8,9,11,12,17,21,22,26,27,28,29]. Some reports suggest that high CRP levels correlate with TB in human patients, but due to a variety of confounding factors, serum CRP levels are insufficient by themselves as biomarker of human disease. We found that CRP-peak levels were significantly different when comparing ATB with LTBI (*p* = 2.30 × 10^−29^, Table 1). Whereas previous studies have always found a significant difference between ATB and LTBI CRP levels, the degree of difference between the CRP values alone is not deterministic of disease progression. 

**Figure 2:**
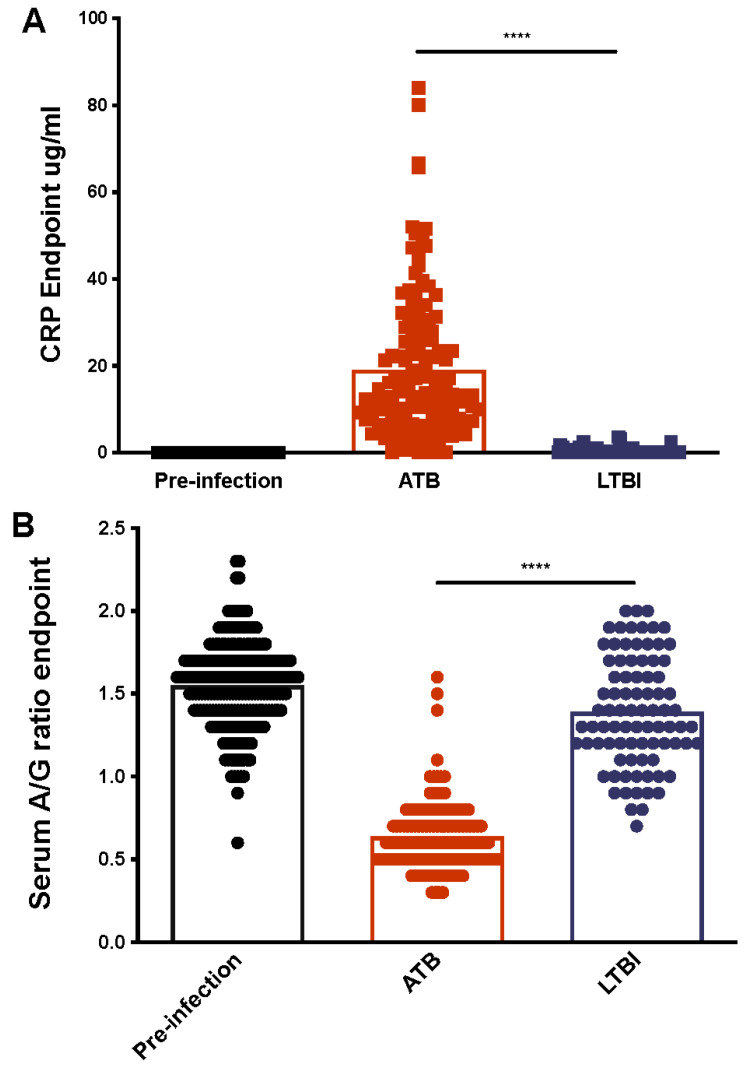
Serum CRP (**A**) and A/G Ratios (**B**) at Endpoint. Endpoint serum CRP values (**A**) and A/G ratios (**B**) were similar between pre-infection (black) and LTBI (blue), but were significantly different between ATB (red) and LTBI. (****, *p*-value of < 0.0001). Each dot represents one animal.

We observed a significant elevation of endpoint serum CRP levels in animals with active TB (n = 134, Figure 2A). In contrast, the levels of endpoint serum CRP remained at or very close to baseline, in animals with LTBI (n = 80, Figure 2A). The difference between pre-infection serum CRP levels and ATB endpoint levels was highly statistically significant when compared (*p ≤* 0.0001). In contrast, the difference between pre-infection values and the endpoint values of animals with LTBI was not significant (*p = 0.859*) (Figure 2A). Highly significant differences were observed between the endpoint serum CRP values of ATB and LTBI animals (*p* = 6.88 × 10^−27^, Table 1, Figure 2A). The levels of peak CRP, regardless of whether they occurred at the endpoint or earlier, were higher in animals which were euthanized due to ATB, relative to animals that developed LTBI (Supplementary Material Figure S1). These results expand on our previously published observations, that serum CRP levels remained at baseline during LTBI in macaques [7]. Following these initial results, we sought to understand if serum CRP levels statistically significantly correlated with disease parameters, such as lung bacterial burden.

We utilized Pearson’s r coefficient analysis to determine significant correlates of bacterial burden (Table 2 and Table 3). Coefficient analysis was also performed on base 10 log of CFU/gm. Calculating the log10(CFU/gm) acted as a form of normalization for easier comparison between CFU/gm values and the measured correlates of disease which had values that were much smaller in magnitude than CFU/gm. CRP peak and endpoint measurements recorded a statistically significant moderate positive correlation to both CFU/gm and CFUlog10 counts for LTBI and ATB cohorts; due to this we looked closer at CRP correlations to other metrics of disease (Table 2 and Table 3). We found that for the ATB group, CRP values correlated negatively to A/G ratio (bottom, endpoint) and lymphocyte percentages (bottom, peak, endpoint); in contrast LTBI and CRP correlated positively to lymphocyte percentages at bottom, peak, and endpoint (Supplementary Data File). For both ATB and LTBI, CRP correlated positively to monocyte and neutrophil percentages. The strength of LTBI and CRP correlations was much weaker than that of ATB, with LTBI having fewer correlations throughout when compared with ATB (Supplementary Data File).

### 2.3. Serum Albumin-to-Globulin (A/G) Ratios Compared between ATB and LTBI

Previous studies have suggested that serum A/G ratio correlates with the development of ATB, but similarly to CRP, it is a non-specific marker of inflammation, and other confounding factors could interfere [30]. A/G ratios were calculated from serum albumin and globulin levels taken prior to infection and necropsy. A significant reduction in A/G ratio, driven by higher serum globulin levels, was observed in animals with active TB (Figure 2B). A/G ratios for macaques with LTBI were only marginally reduced. The differences in endpoint A/G ratios for the ATB and the LTBI groups were statistically significant (*p* = 3.43 × 10^−36^, Table 1, Figure 2B). The A/G ratios for animals with ATB ranged from 0.3–1.6 with mean values of 0.64, whereas the corresponding ratios for animals with LTBI were between 0.7–2.0 with mean values 1.4. The mean ratio for naive animals was 1.56 (range 0.6–2.3). The reduction in A/G ratio during ATB was highly statistically significant relative to pre-infection values (*p* < 0.0001). The endpoint A/G levels for animals with LTBI were themselves statistically significant in that they were different from the baseline (pre-infection) samples (*p* < 0.01). This finding allows us to differentiate LTBI from naïve or healthy individuals; however, this can also be triggered by mild inflammations and other opportunistic infections, and therefore, an antigen specific test is necessary for confirmation. We also observed a statistically significant difference between ATB and LTBI for A/G ratios at peak and bottom concentrations (*p* = 0.001, 5.43 × 10^−28^, Table 1). Given the significant differences found between disease conditions, A/G ratios, and CRP levels, we hypothesized that a correlation between the lung specific data from *Mtb* infection and clinical data that can be obtained from blood samples would better differentiate between active and latent infection. We found that A/G ratio bottom and endpoint values negatively correlated to CFU/gm and (CFU/gm)log10 for LTBI but only showed significant correlation in ATB group when correlated to (CFU/gm)log10 (Table 2 and Table 3). Based on these results we performed regression analysis to determine the predictive capabilities of our most relevant correlates.

**Figure 3:**
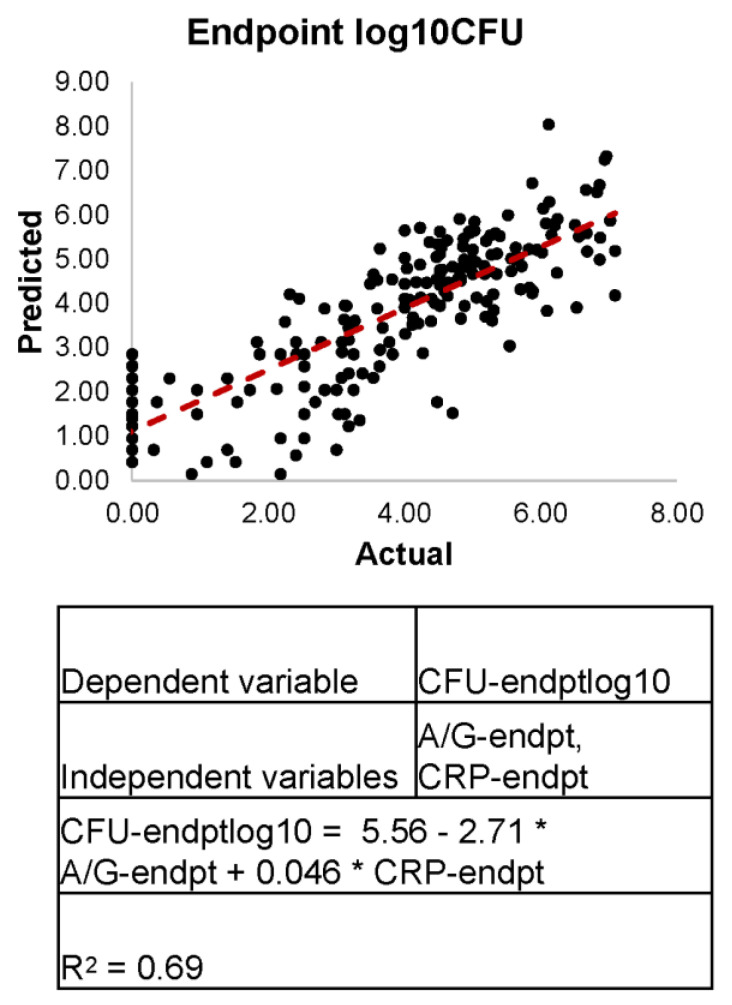
Linear Regression Models for Predicting log10CFU Counts at Endpoint. Utilizing multilinear regression analysis, we found that CRP at endpoint and A/G ratio at endpoint were able to accurately predict log10CFU counts at endpoint, allowing us to determine if the host was actively or latently infected.

Linear regression analysis was performed using a combined dataset of LTBI and ATB. We found that log10(CFU/gm) as our dependent variable was a relatively more accurate predictor of disease outcome when compared with CFU/gm. The poor predictive power of CFU/gm was seen due to the small magnitude of independent variable values coupled with the larger magnitude values from CFU/gm, which was the dependent variable; therefore, the log10(CFU/gm) was utilized. We found the A/G ratios and CRP levels taken at endpoint are moderately able to predict log10(CFU/gm) with an R-squared value of 0.69 (Figure 3). The addition of endpoint monocyte, lymphocyte, and/or neutrophil counts as dependent variables increased the predictive power from a range of R-squared 0.69 ± 0.0059. The minimum and most predictive independent variables needed to predict disease outcome was determined to be the A/G ratios and CRP levels. These results further support our initial observations, that A/G ratio and CRP values can predict host disease state.

### 2.4. Myeloid and Lymphoid Cell Counts Compared between ATB and LTBI

**Figure 4:**
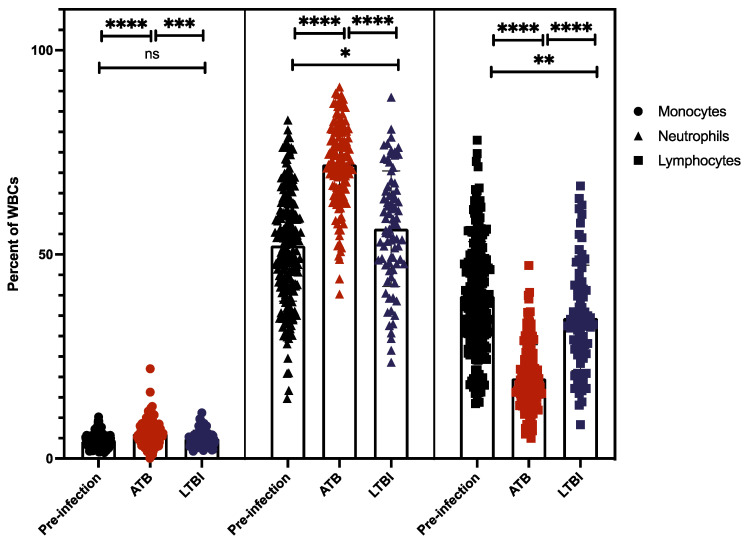
Percentage of specific WBCs at Endpoint, of complete blood cell counts. Endpoint cell counts between ATB (red) and LTBI (blue) were different across all cell types measured. (ns, *p*-value of > 0.05; *, *p*-value of ≤ 0.05; **, *p*-value of ≤ 0.01; ***, *p*-value of ≤ 0.001; ****, *p*-value of < 0.0001).

Myeloid cells, such as monocytes and neutrophils, act as both sites of replication for the *Mtb* bacilli and are responsible for the release of CRP and changes in A/G ratio. Lymphocytes are thought to be a strong correlate of protection from *Mtb* infection and are essential for clearance [31]. Given the important role of each cell type in the immune response to *Mtb* infection, we studied monocytes, neutrophils, and lymphocytes accumulating in the peripheral blood as a percentage of all blood cells, in the animals with the two disparate clinical outcomes (Figure 4, Supplementary Figure S2). 

*Lymphocytes.* We observed distinct differences in lymphocyte percentages between pre-infection and ATB samples as well as between ATB and LTBI. The percentage of lymphocytes in the peripheral blood of uninfected animals ranged from 13.8–78.0%, with a mean value of 40%, whereas animals at endpoint with ATB exhibited values ranged from 6.0–47.3% with a mean of 20%, and LTBI values ranged from 8.3–66.8% (range) with a mean of 34.5%. The differences between lymphocyte percentage at pre-infection and at LTBI endpoint were only marginally significant (*p* = 0.01), but the differences between pre-infection and ATB were found to be far more significant (*p* = 0.0001, Figure 4, Supplementary Figure S2). The bottom and endpoint lymphocyte percentages in animals with ATB were very significantly different from LTBI (*p* = 2.44 × 10^−07^, 2.40 × 10^−15^, Figure 4, Table 1). We found that the percentage of lymphocytes in the blood of macaques trended towards lower percentages for ATB animals when compared with pre-infection, whereas LTBI stayed roughly the same when compared with pre-infection (Figure 4, Supplementary Figure S2). Correlation analysis revealed that lymphocyte bottom and endpoint percentages positively correlated with CFU/gm, and (CFU/gm)log10 counts for LTBI and ATB NHPs, though the significance and correlation were marginal (*p* < 0.01, Table 2 and Table 3). 

*Monocytes.* The percentage of monocytes present in blood prior to infection ranged from 1.5–10.2%, with a mean value of 4.3% (Figure 4, Supplementary Figure S2). Overall monocyte values for both disease states maintained a similar trend; ATB had a range of 0.1–22% and a mean of 6.21%, whereas LTBI had a range of 1.8–11.2% and a mean of 5% (Figure 4). The differences between pre-infection and the ATB values were marginally significant (*p* = 0.02); however, the differences between pre-infection and LTBI (*p* = 0.11) were not significant. For both bottom and endpoint values, ATB and LTBI were statistically significantly different (*p* = 0.045, 0.001, Figure 4, Table 1). Overall, our results show that no significant changes in monocyte levels occur when comparing pre-infection to LTBI, in contrast ATB experiences a marginal increase in the percentage of monocytes. Further analysis showed that monocyte peak and endpoint counts positively correlated to CFU/gm counts for the LTBI group only, and correlation was lost when transformed into base 10 logarithm, referred to as (CFU/gm)log10 (Table 2 and Table 3). 

*Neutrophils.* Similar to lymphocytes, neutrophil percentages in the peripheral blood display a distinct trend in ATB primate group. The mean pre-infection value for neutrophils was 52.2%, with a range of 14.7-83%. During ATB infection, the endpoint mean for animals increased to 72% with a range of 40.3–91%; this increase was highly statistically significant from pre-infection (*p* < 0.0001, Figure 4, Supplementary Figure S2). The mean endpoint value for animals with LTBI was 56.3%, with a range of 23.6–88.5%. The percentage of neutrophils at the LTBI endpoint was marginally different when compared with pre-infection (*p* = 0.012). When comparing LTBI and ATB at endpoint and peak, we found significant differences between the two groups (*p* = 2.03 × 10^−14^, 3.38 × 10^−06^, Figure 3, Supplementary Figures S2 and S3, Table 1). Our results show that ATB was characterized by significantly higher percentages of neutrophilia in the peripheral blood as compared with LTBI (Figure 4). On the contrary, the percentages of lymphocytes were significantly lower in the ATB, relative to the LTBI group. in conjunction with lower lymphocyte percentage (Figure 4). Similar to monocyte correlations, neutrophil peak and endpoint percentages had a positive correlation with CFU/gm for ATB only (Table 2 and Table 3). Overall, we found the LTBI cell population correlations to be more tenuous when compared with the strength of the ATB correlations, with LTBI correlations having less statistical significance and frequency.

**Figure 5:**
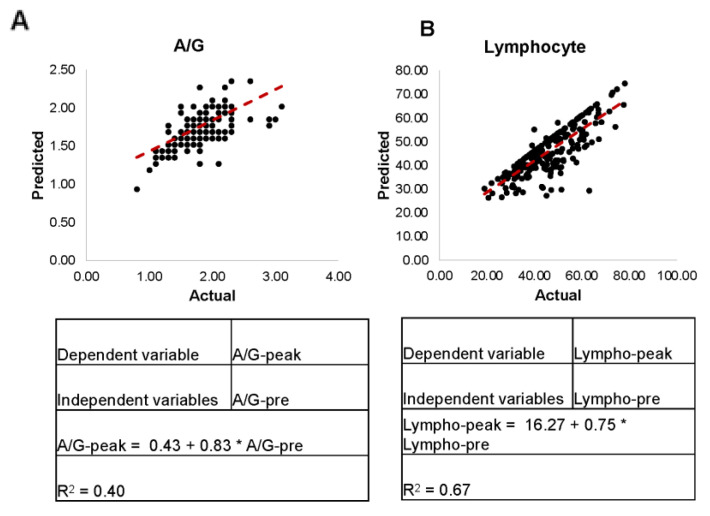
Linear Regression Models for A/G Ratio (**A**) and Lymphocyte Percentages (**B**) at Peak. Utilizing univariate linear regression, we determined that for A/G ratio (**A**) and lymphocyte percentages (**B**) at peak, pre-infection values could be used to determine post-infection response.

Through our examination of our correlation matrix, we identified strong correlations between pre-infection and post-infection values for A/G ratio and lymphocyte percentages in both ATB and LTBI groups (Supplementary Data File). Having observed this relationship, we applied a univariate linear regression to determine if pre-infection was deterministic of post-infection. Though all combinations were analyzed, we found that pre-infection most accurately predicted peak values for A/G ratio (R^2^ = 0.40, Figure 5A) and lymphocyte percentage (R^2^ = 0.67, Figure 5B), although these correlations are of moderate strength at best. For monocyte (R^2^ = 0.41) and neutrophil (R^2^ = 0.55) percentages, pre-infection best predicted bottom values, though the power of the regression was significantly weaker than that of the lymphocyte regression.

**Figure 6:**
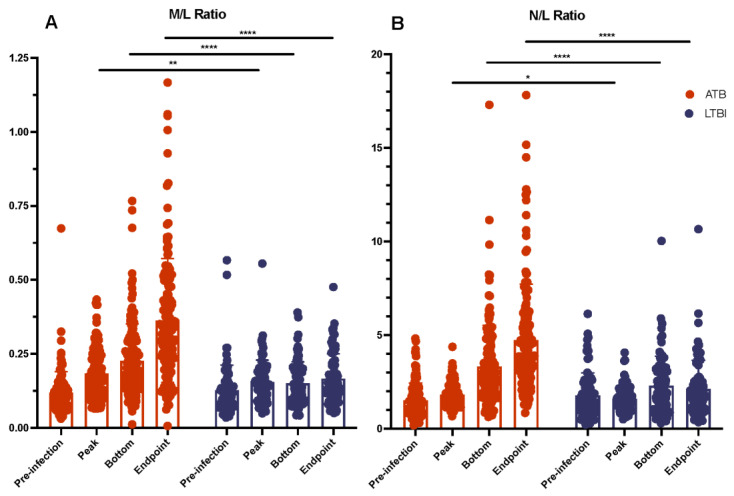
Monocyte-to-Lymphocyte (**A**) and Neutrophil-to-Lymphocyte (**B**) Cell Ratios. Cell ratios were utilized as a simple, standardized biomarkers that were able to differentiate between ATB and LTBI. (*, *p*-value of ≤ 0.05; **, *p*-value of ≤ 0.01; ****, *p*-value of < 0.0001).

*Cell ratios*. Ratios of monocytes-to-lymphocytes (M/L) and neutrophils-to-lymphocytes (N/L) were compared between active and latent infections. Ratios were calculated using cell identifiers (bottom, peak, endpoint) for both the numerator and denominator of the ratio; for instance, the M/L peak was calculated by dividing monocyte peak value by lymphocyte peak value. Presenting the ratios in this manner allows us to emphasize the maximal magnitude of monocyte and neutrophil influxes, as compared with lymphocytes, as well as overcome the difference in sample timings across such a large sample size. Cell ratios are often used as a metric for determining the severity of infection from peripheral blood and the risk of disease progression in latent TB cases [32,33,34]. We found that for both M/L and N/L ratios, ATB and LTBI were significantly different at peak, bottom, and endpoint measures (Figure 6). M/L and N/L ratios were significantly higher in ATB than LTBI for all measures except pre-infection. Although LTBI ratios remained consistently the same from pre-infection to endpoint, ATB ratios fluctuated upwards from pre-infection to endpoint (Figure 6). This increase in M/L and N/L ratio values found in ATB, but not LTBI, is due to high lymphocyte percentages and low monocyte and neutrophil percentages in LTBI when compared with ATB (Figure 6). This data agrees with previous findings and supports claims that M/L and N/L ratios can be versatile tools for diagnosis or treatment [35,36].

**Figure 7:**
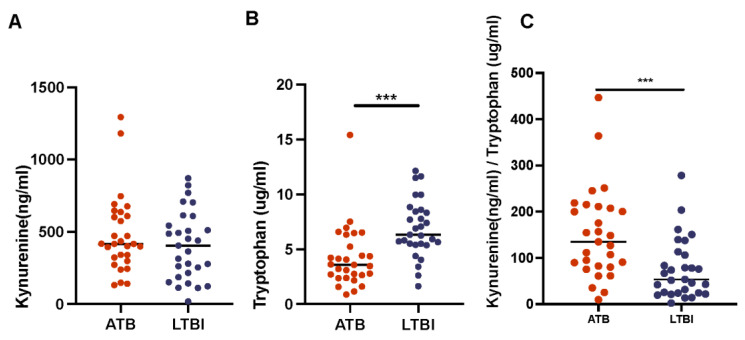
Kynurenine-to-Tryptophan Ratio. ELISA was performed to determine the levels of kynurenine (**A**), and Tryptophan (**B**), and through these calculate kynurenine-to-tryptophan ratio (**C**), allowing us to extrapolate the amount of the enzyme, IDO, which converts tryptophan to kynurenine within the cell. (***, *p*-value of ≤ 0.001).

*Kynurenine and Tryptophan Ratios*. Having established that the levels of myeloid cells (monocytes as well as lymphocytes) are increased in the peripheral blood as a function of active disease, we next studied the expression of known markers of myeloid cell immune suppression in the peripheral blood on a subset of each group, LTBI (n = 29) and ATB (n = 29). We found that kynurenine-to-tryptophan ratios were a strong determinant of active TB infection (Figure 7). Previous studies have found that K/T ratio increased in a bacterial burden–dependent manner, due to increased production and availability of IDO (indoleamine 2,3-dioxygenase) [26,37]. IDO is an enzyme that catabolizes tryptophan to kynurenine, and this enzyme is primarily expressed by professional antigen presenting cells [38]. The host initially increases IDO in effort to starve the tubercule bacillus of tryptophan, an essential amino acid for the bacillus, but *Mtb* is capable of synthesizing its own tryptophan when under stress [39,40]. Our study found that the levels of tryptophan in ATB host’s plasma was significantly lower than that of LTBI, but that there was not a cognate, significant difference in kynurenine availability between the two groups (Figure 7, Table 1). As a result, K/T ratios were significantly higher for ATB relative to LTBI samples (Figure 7C, Table 1). These results confirm our earlier finding that during active TB infection, there are increased amounts of IDO present in the periphery. To further quantify the relationship between bacterial burden and K/T ratio we performed correlation analysis. We found that K/T ratios were significantly positively correlated to CFU/gm for both ATB and LTBI (Table 2 and Table 3). ATB was observed to have moderate positive correlations to (CFU/gm)log10 for both kynurenine and K/T ratio, whereas LTBI had positive correlations to CFU/gm and log10CFU/g for K/T ratios only (Table 2 and Table 3, Figure 7). The absence of a significant correlation between tryptophan and bacterial load indicates that the likely driving force behind the correlation between K/T ratio and host disease state is the rate-limiting enzyme in the tryptophan catabolism pathway—IDO. It appears, based on these results, that the correlative relationship between IDO and bacterial load is further strengthened as the bacterial load increases, as evidenced by a lack of correlation between kynurenine and LTBI bacterial load, and as a correlation kynurenine is present in ATB. Based on this analysis and other similar works, IDO inhibitors are currently being investigated as possible host directed therapies for TB [26]. 

**Figure 8:**
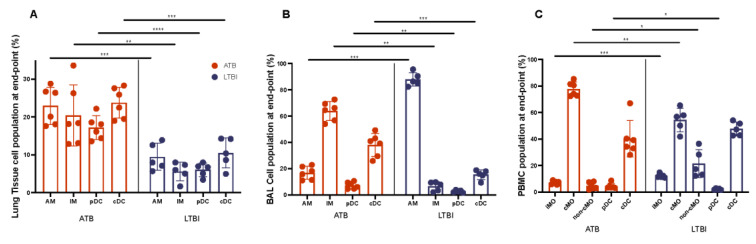
Myeloid Cell Populations. Cell populations were determined using flow cytometry in lung (**A**), BAL cells (**B**)_and PBMCs (**C**) from a subset of macaques with ATB and LTBI, and a full gating strategy is available in supplementary Figures S4 and S5. AM (alveolar macrophages), IM (interstitial macrophages), pDC (plasmacytoid dendritic cells), cDC (conventional dendritic cells), iMo (intermediate monocytes), cMo (classical monocytes), and non cMo (Non-classical monocytes). (*, *p*-value of ≤ 0.05; **, *p*-value of ≤ 0.01; ***, *p*-value of ≤ 0.001; ****, *p*-value of < 0.0001).

*Myeloid Population*. Given the strength of the differences within the myeloid population seen in our data (Figure 3 and Figure 6, Supplementary Figure S2), we decided to perform flow cytometry on a representative subset of animals, ATB (n=6) and LTBI (n=5). Lung tissue, bronchoalveolar lavage cells, and peripheral blood were processed to single-cell suspension and analyzed using flow cytometry. Flow cytometry analysis was conducted by gating first on live leukocytes using the Live/Dead stain and CD45, then lymphocytes were gated out by using CD3 and CD20 lineage markers. The remaining cells were gated on HLA-DR+ subset for the selection of myeloid subsets: CD163+ 206- interstitial macrophages (IM) and CD163+ 206+ alveolar (AM) macrophages, CD163- CD123+ plasmacytoid (pDC), and CD163- CD11C classical (cDC) dendritic cells. In PBMCs, the HLA-DR+ subset was gated on CD14 and CD16 to identify CD16+ non-classical (non-cMo), CD14+ classical (cMo), and CD14+CD16+ inflammatory (iMo) monocytes, followed by CD123+ (pDC) and CD11C+ gating on the double negative CD14-CD16- subset. We found distinct myeloid populations in lung tissue, BAL, and the periphery (Figure 8, Supplementary Figures S3 and S4). Similar to our previously published findings, significant remodeling of myeloid populations was observed between ATB and LTBI animals [41]. In both lung tissue and BAL cells, in the case of all except BAL AM (alveolar macrophages), ATB cell percentages significantly exceed that of LTBI (Figure 8A, Table 4). The majority of cells in lung tissue were AMs and cDCs (conventional dendritic cells), with a range of 18.1–28.8%, 19.0–28.3% for ATB, and 5.71–13.90, 4.97–14.3% for LTBI, respectively. In BAL cells, the population was predominantly IM (interstitial macrophages) and cDC for ATB, and AM and cDC for LTBI (Figure 8B). Similar to lung tissue, IM and cDC percentages in ATB BAL significantly exceeds that of LTBI. In BAL, IM cells range from 2.21–9.77% in LTBI and 11.1–22.9 in ATB. cDC cells range from 9.5–18.9% in LTBI and 26–49.3% in ATB. In BAL, AM cells range from 82.4–95.6% in LTBI and 54.1–72.7% in ATB. We found that the PBMC population was majority cMo (classical monocytes) and cDC (Figure 8C). In LTBI animals, cMo and cDC cells ranged from 41.9–65.40% and 42.70–54.10%, respectively. Although ATB animals saw a significant increase in average cMo, and a lower average cDC in PBMCs which ranged from 72.40–85.30% and 28.10-67.00%, respectively (Figure 8C, Table 4), the increase in periphery cMo is significantly positively correlated to increasing iMo in ATB; however, in LTBI, cMo is significantly negatively correlated to non-cMo (Supplementary Data File). This indicates a change in relationship between cell populations based on infection state.

All PBMC and myeloid subpopulations were significantly different with the exclusion of cDC, in which Welch’s t test results were skewed slightly by a single outlier due to a smaller group size (Table 4). There was a distinct shift in magnitude of cell populations when comparing ATB with LTBI, and when comparing the relationship between cell types within each condition, as seen in the shift in BAL cells between AM and IM. Overall, we observed an acute change in peripheral and lung cell profile, with an observed influx of myeloid cells to the alveolar space from the periphery. Based on these results, further correlation analysis was performed, which showed that LTBI lung alveolar macrophages (Lung.AM) were strongly negatively correlated to (CFU/gm)log10 (Table 3, Figure 8A). This is in contrast to visual interpretations which show ATB primates, which carry a much higher bacterial load than LTBI, having increased amounts of AMs in comparison to LTBI. This unique correlation between alveolar macrophages is quite significant and has a strong negative correlation coefficient (Table 3, Figure 8A). Lung alveolar cells also had a negative correlation to K/T ratio for ATB animals but not LTBI (*p* = 0.012, Pearson’s r = −0.91, Supplementary Data File). Given our sample size we will need to further explore the implications of these relationships in the future.

## 3. Methods

### 3.1. Study Design

To establish a reference peripheral biomarker profile of ATB vs LTBI in the RM model of TB we performed a retrospective observational analysis based on a larger cohort of Indian-origin rhesus macaques (Macaca mulatta) that were experimentally infected with Mtb by our group either CDC1551 (n = 207), Erdman (n = 12), or H37Rv (n = 6) strains during 2007–2014. Peripheral biomarkers originating from serum biochemistry and complete blood count were correlated with lung pathogenic burden and pathology to draw correlations which were then validated using cryopreserved samples. 

### 3.2. Data Acquisition 

Clinical data stored in the Animal Records System (ARS) at the Tulane National Primate Research Center (TNPRC) was utilized for this study. This data was specific to animals assigned to the various projects in either the Kaushal or the Mehra labs. Animals were chosen if they were experimentally infected with *Mtb* (either CDC1551, Erdman or H37Rv strains). Animals had been infected with *Mtb* as if they were specific pathogen-free and mycobacteria-naïve. These infections occurred between the years of 2007–2014. We have excluded data from animals coinfected or treated for disease. Methods for quantification of lung bacterial burdens (CFU/gm) and pathology have been described earlier [4,5,6,7,8,9]. Bacterial burden and immunologic response were determinants for classification to either active (ATB, n = 140) or latent (LTBI, n = 85) infected groups. Animals with consecutively (three weeks) higher than normal serum CRP levels, higher than a score of 1 on chest X-rays, or the presence of viable *Mtb* bacilli in BAL were deemed to have ATB. Animals were deemed to have LTBI if they tested positive for tuberculin skin after experimental *Mtb* infection without radiological and microbiological evidence of TB. Through the use of serum chemistry, serum CRP levels and A/G ratios were obtained, using a Beckman Coulter AU 480 fully automated chemistry analyzer. Blood cell levels for monocytes, lymphocytes, and neutrophils were also quantified as percentages of complete blood cell counts (EDTA blood) from the animals at that time-point. For validation, cryopreserved samples were used to enumerate cell fractions by flow cytometry and estimate Kynurenine and Tryptophan (K/T) ratio in plasma, methods for which are described in detail in previous publications [4,5,6,7,8,9]. Post-infection minimal and maximal values observed throughout the experiment until necropsy were denoted as bottom and peak, respectively, and used throughout the data processing.

### 3.3. Kynurenine and Tryptophan Ratios in Plasma

A subset of NHPs from both ATB (n = 29) and LTBI(n = 29) groups were selected to evaluate the enzymatic activity of indoleamine 2,3-dioxygenase (IDO) in plasma through the observation of its substrate and product. We used a kynurenine and tryptophan ELISA kit (Immusmol, ISE-2227). The lower range of sensitivity of detection for kynurenine and tryptophan were between <47.5 ng/mL and <2.5 μg/mL, respectively, and K/T ratios calculated. 

### 3.4. Flow Cytometry

Flow cytometry was performed on lung tissue, whole-blood, and BAL (bronchoalveolar lavage) cell samples obtained from a subset of 11 animals, using methods previously described in earlier publications [6,9]. For myeloid phenotyping, the following antibodies were used: Live/Dead FVS440, CD45 BUV396 (D0581283), HLA-DR APC-Cy7(L243), CD3 Alexa fluor (SP34-2), CD206 BV421(19.2), CD163 PE(GHI/61), CD123 PerCP-Cv5-5(7G3), CD11c BV711(3.9), CD16 (BV480), and CD14 APC (M5E2). All were purchased from BD Biosciences (San Jose, CA, USA). Flow cytometry analysis was conducted by gating first on live leukocytes using Live/Dead stain and CD45, then lymphocytes were gated out by using CD3 and CD20 lineage markers. The remaining cells were gated on HLA-DR+ subset for the selection of myeloid subsets: CD163+ 206- interstitial macrophages (IM) and CD163+ 206+ alveolar (AM) macrophages, CD163- CD123+ plasmacytoid (pDC), and CD163- CD11C classical (cDC) dendritic cells. In PBMCs, HLA-DR+ subset was gated on CD14 and CD16 to identify CD16+ non-classical (non-cMo), CD14+ classical (cMo), and CD14+CD16+ inflammatory (iMo) monocytes, followed by CD123+ (pDC) and CD11C+ gating on a double negative CD14-CD16- subset.

### 3.5. Difference Analysis

Welch’s t tests and ordinary one-way ANOVA were performed to compare ATB (n = 140) and LTBI (n = 85) datasets. T-tests were run on CFU/gm and (CFU/gm)log10 counts obtained from lung at endpoint as well as on CRP values. A/G ratio, Lymphocyte, Monocyte, and Neutrophil percentages at pre-infection, peak, bottom, and endpoint were compared between ATB and LTBI. Pre-infection sampling was conducted one or two weeks prior to experimental *Mtb* infection and are indicative of healthy TB naïve animals. The endpoint was governed by experimental protocol unless the clinical state of the NHP required humane euthanasia based on pre-specified endpoint criteria that were evaluated and decided by one or more board-certified veterinary clinicians. Peak and bottom levels were based on obtained longitudinal data. Welch’s t tests were performed using R with RStudio interface and ordinary one-way ANOVA using GraphPad Prism 8. The Shapiro–Wilk test was performed to ascertain the normality of the data.

### 3.6. Correlation

Pearson correlation analysis was performed using R programming language with RStudio as interface. Post and pre-infection, LTBI and ATB datasets were analyzed. To fully demonstrate quantifiable differences between ATB and LTBI disease states, analysis was performed within either the LTBI or ATB dataset, not on an aggregate dataset. We observed distinct correlative patterns that were present regardless of level of bacterial burden and correlative patterns isolated to individual disease states. 

### 3.7. Linear Regression

Using R and the Excel extension StatPlus we performed linear regression analysis of our data with the purpose of determining which biomarkers would best predict the outcome of disease. We utilized an aggregate dataset of both LTBI and ATB, unlike correlation analysis which were performed on separated LTBI and ATB datasets. For this analysis, it was important that a single regression predict bacterial burden regardless of disease classification. Statistically significant correlations were used to direct which independent variables were best suited to generate CFU/gm predictive linear regressions. Multilinear and univariate linear regressions were applied utilizing all metrics as independent variables with an emphasis on R-squared as the deterministic factor. 

## 4. Discussion

Previous human studies have found that C-reactive protein [42,43,44], and A/G ratio [45] are differentially expressed in ATB patients when compared with LTB. Our current results, obtained using the human-like NHP model of TB, support these earlier findings. This is unsurprising given that we have previously demonstrated a relationship between serum CRP levels and TB progression in NHPs’ progression [6,7,8,9,11,12,17,21,22,26,27,28,29]. LTBI values for both CRP and A/G ratios remained at similar levels to that of pre-infection, whereas in animals with ATB, there were elevated levels of serum CRP and a lowered A/G ratio when compared with LTBI. Previous studies have found that in active TB disease cases in human beings, albumin levels decrease whereas globulin levels increase, leading to low A/G ratios [43]. We also observed a higher K/T ratio in ATB than in LTBI. This last aspect is particularly interesting. We have previously shown [26] that IDO expression is induced in macrophages of both rodent and primate origin immediately after *Mtb* infection. IDO is chiefly catabolic in function and leads to the generation of kynurenine. Tryptophan is an essential amino acid for most pathogens, and therefore, the immune mechanism to induce IDO and starve the pathogen of such an essential nutrient is successful [46]. In the case of *Mtb*, however, Zhang et al. have shown that this remarkable pathogen is able to anabolize its own tryptophan [40]. Furthermore, we have shown that the tryptophan biosynthetic machinery is induced in NHP TB granulomas, indicating that tryptophan is produced by intra-granulomatous *Mtb* [21]. For this reason, the immune strategy to starve *Mtb* of tryptophan is unsuccessful. Additionally, tryptophan is required for the rapid proliferation of T cell responses, and the high IDO expression precludes this, as shown in Gautam et al. IDO is transcriptionally regulated by both Type I (α, β) and Type III (γ) interferons in a context dependent manner [47]. Both types of interferon signaling pathways are induced by *Mtb* infection, likely leading to the induction of IDO (and a high K/T ratio in ATB) during *Mtb* infection. Our earlier work first identified that IDO was intensely expressed in the TB granuloma [13,20,26]. More recently, we have conclusively shown that the majority of IDO mRNA expression in the host granuloma occurs on interstitial, inflammatory, IFN-responsive macrophages that are recruited to the lung post-infection [41] The majority of these cells are present in the inner myeloid ring of the lesion. More recently, our investigations into the role of Myeloid-derived Suppressor Cells (MDSCs) during experimental *Mtb* infections in macaques has revealed that IDO is also expressed in these cells at the granuloma periphery, although the majority of IDO expression occurs on interstitial macrophages. Studies from human samples from TB patients and household contacts also show the recruitment of MDSCs to the lungs [48,49,50]. Clearly, spatial expression of IDO in the various regions of the granuloma is an important event that promotes *Mtb* persistence. These results are now supported by high-dimension, multiplexed imaging studies of human granulomas, which are characterized by high levels of expression of two immunoregulatory proteins IDO and PD-LI [51]

Serum CRP levels, A/G ratios, and K/T ratios are influenced heavily by the recruitment of myeloid cells. Our results may also indicate massive neutrophil accumulation in the periphery and myeloid cellular influx from periphery to lungs in animals with ATB, relative to LTBI. It has recently been shown using single cell technologies, that myeloid cells recruited to the lung may promote inflammatory, interferon (IFN) responses [41]. In conjunction, K/T ratios have been shown to cause increased T regulatory cells and decreased T effector memory cells which may impair host response during active infection [52]. More recently, we have shown in the NHP model of *Mtb*/SIV co-infection, that myeloid cell turnover is directly correlated with uncontrolled immune activation and reactivation of TB [28,53,54]. A recent multiplexed imaging study also indicates that expression of IDO on myeloid cells is a key feature of human TB granulomas [51]. Taken together, our results could suggest that myeloid mediated inflammation may be a key event in active TB that could be further leveraged to develop human-specific TB biomarkers. If validated in a human clinical setting, these results may have the potential to simplify detection of TB disease, thus providing rapid access to the patients that need it the most. These results could also be applied to future NHP studies as a non-invasive way to monitor host disease status.

Macaques with LTBI exhibited a higher percentage of lymphocytes, a lower percentage of neutrophils, and a slightly lower percentage of monocytes in the blood. We found that a higher bacterial burden, as experienced by NHPs with ATB, resulted in a greater influence over host response. This was evident in ATB correlations, which were more frequent, as well as significant, when compared with LTBI correlations. Through our exploration of this correlative pattern, we were able to generate two linear regressions in which pre-infection values were able to predict post-infection values (A/G ratio, lymphocyte percentages). The predictive capabilities of pre-infection disposition will require more exploration in the future with a larger dataset, but it is a very interesting finding nonetheless. Our lower powered regressions, which predicted bacterial load through the use of total BAL cells, total PBMCs, and BAL cDCs, indicated a possible strong relationship that will need a larger sample size to explore more thoroughly. CRP values and A/G ratios have been used as an indicator of infection and inflammation. Utilizing multilinear regression guided by correlation, we were able to use CRP and A/G ratio values and predict bacterial burden. Due to the similarity between pre-infection and LTBI values, an accurate distinction between the two host states is difficult and may require additional or different biomarkers. Though the distinction between LTBI and pre-infection was imprecise, the differences between LTBI and ATB were clearer and could be characterized through multilinear regression. This regression may be expanded in the future with the potential addition of larger datasets and different biomarkers, as well as its application for new and foreign datasets as a predictive formula.

Overall, we observed correlational changes in cell percentage, CRP, A/G ratio, and K/T ratio. We have characterized cell subsets of the periphery and the lung, and highlighted those differences found between ATB and LTBI. These results may allow for the development of a unique pattern of correlates, which could accurately predict the host disease state between ATB and LTBI. These correlations may have strong potential application for both future NHP and human clinical studies. One potential limitation of our study is that most of the differences that we documented mark the acute form of the disease, which has other manifestations that can be clinically measured; however, growing evidence suggests that *Mtb* infection is not only characterized by two extremes of LTBI and ATB, but that it represents a spectrum. As such, identification of preliminary pathways and segments of the immune system, which can be used to differentiate such specific outcomes of infection, is important. NHP models can play an important role in this since they recapitulate many aspects of the human TB syndrome.

## Figures and Tables

**Table 1:** Welch’s t Test. Empty spaces indicate differences were not statistically significant.

Welch’s *t* Test ATB v. LTBI	
	*p*-Value
CFU	4.48 × 10^−06^
CFU-log10.	3.31 × 10^−44^
CRP-pre	
CRP-peak	2.30 × 10^−29^
CRP-endpt	6.99 10^−27^
A/G-pre	
A/G-peak	0.001
A/G-bottom	5.43 × 10^−28^
A/G-endpt	3.43 × 10^−36^
Lympho-pre	
Lympho-peak	
Lympho-bottom	2.44 × 10^−07^
Lympho-endpt	2.40 × 10^−15^
Mono-pre	
Mono-peak	
Mono-bottom	0.045
Mono-endpt	0.001
Neutro-pre	
Neutro-peak	3.38 × 10^−06^
Neutro-bottom	
Neutro-endpt	2.03 × 10^−14^
L/M-pre	
L/M-peak	0.012
L/M-bottom	0.004
L/M-endpt	0.013
N/M-pre	
N/M-peak	
N/M-bottom	
N/M-endpt	
N/L-pre	
N/L-peak	0.021
N/L-bottom	9.27 × 10^−05^
N/L-endpt	2.02 × 10^−15^
M/L-pre	
M/L-peak	0.007
M/L-bottom	2.48 × 10^−08^
M/L-endpt	8.62 × 10^−19^
Kynurenine	
Tryptophan	2.82 × 10^−04^
K/T	9.21 × 10^−04^

**Table 2:** ATB CFU and log10CFU Pearson Correlations. Empty spaces indicate differences were not statistically significant. #N/A: not available (for correlation to self value).

A. ATB CFU Correlations			B. ATB log_10_CFU Correlations		
	*p*-Value	Coefficient		*p*-Value	Coefficient
CFU-endpt	#N/A	#N/A	CFU-endpt	0.000	0.671
CFU-endptlog10	0.000	0.671	CFU-endptlog10	#N/A	#N/A
CRP-pre			CRP-pre	0.004	−0.243
CRP-peak	1.43 × 10^−08^	0.458	CRP-peak	2.17 × 10^−12^	0.549
CRP-endpt	2.72 × 10^−08^	0.450	CRP-endpt	9.66 × 10^−14^	0.576
A/G-bottom			A/G-bottom	0.024	−0.192
A/G-endpt			A/G-endpt	0.003	−0.250
Lympho-bottom	0.014	−0.209	Lympho-bottom	0.014	−0.208
Lympho-endpt	0.013	−0.212	Lympho-endpt		
Mono-peak			Mono-peak		
Mono-endpt			Mono-endpt		
Neutro-peak	0.025	0.191	Neutro-peak		
Neutro-endpt	0.012	0.213	Neutro-endpt		
LM-endpt	0.037	0.178	LM-endpt		
NM-bottom	0.029	0.186	NM-bottom		
NM-endpt	0.012	0.212	NM-endpt		
ML-pre	0.036	0.178	ML-pre	0.045	0.170
kyn_ng_ml			kyn_ng_ml	0.014	0.453
K_T_ng_ug			K_T_ng_ug	0.027	0.411
kyn_ug_ml			kyn_ug_ml	0.014	0.453
K_T			K_T	0.027	0.411
Lung.AM			Lung.AM		

**Table 3:** LTBI CFU and log10CFU Pearson Correlations. #N/A: not available (for correlation to self value).

A. LTBI CFU Correlations			B. LTBI log_10_CFU Correlations		
	*p*-Value	Coefficient		*p*-Value	Coefficient
CFU-endpt	#N/A	#N/A	CFU-endpt	1.26 × 10^−13^	0.703
CFU-endptlog10	1.26 × 10^−13^	0.703	CFU-endptlog10	#N/A	#N/A
CRP-pre	0.044	0.224	CRP-pre		
CRP-peak	0.024	0.251	CRP-peak	0.001	0.365
CRP-endpt	0.025	0.248	CRP-endpt	0.028	0.244
A/G-bottom			A/G-bottom	0.015	−0.268
A/G-endpt	4.89 × 10^−04^	−0.377	A/G-endpt	3.90 × 10^−04^	−0.383
Lympho-bottom			Lympho-bottom		
Lympho-endpt			Lympho-endpt		
Mono-peak	0.034	0.234	Mono-peak		
Mono-endpt	0.050	0.217	Mono-endpt		
Neutro-peak			Neutro-peak		
Neutro-endpt			Neutro-endpt		
LM-endpt			LM-endpt		
NM-bottom			NM-bottom		
NM-endpt			NM-endpt		
ML-pre			ML-pre		
kyn_ng_ml			kyn_ng_ml		
K_T_ng_ug	0.043	0.385	K_T_ng_ug	0.010	0.480
kyn_ug_ml			kyn_ug_ml		
K_T	0.043	0.385	K_T	0.010	0.480
Lung.AM			Lung.AM	0.004	−0.978

**Table 4:** Welch’s t Test Myeloid Cell Populations.

Welch’s T Test ATB v. LTBI			
*p*-Value			
	Lung	BAL	PBMC
AM	5.44 × 10^−04^	1.35 × 10^−04^	
IM	5.23 × 10^−03^	3.27 × 10^−03^	
pDC	7.58 × 10^−05^	5.26 × 10^−03^	3.99 × 10^−02^
cDC	4.24 × 10^−04^	6.99 × 10^−04^	
iMO			5.32 × 10^−04^
cMO			1.79 × 10^−03^
non-cMO			2.14 × 10^−02^

## Data Availability

Data is available at GPL10183.

## Supplementary Materials

The following supporting information can be downloaded at: https://www.mdpi.com/article/10.3390/pathogens11050544/s1, Figure S1: CRP values at Pre-Infection, Peak, and Endpoint; Figure S2: Pre-infection and Endpoint Cell Percentages; Figure S3: Flow cytometry gating strategy for PBMCs; Figure S4: Flow cytometry gating strategy for BAL and Lung tissue cells. Also included is the Supplementary Data File, an .xlsx file that contains all correlations performed.
