# Supplementary material for: Peripheral Blood Markers Correlate with the Progression of Active Tuberculosis Relative to Latent Control of Mycobacterium tuberculosis Infection in Macaques
Source: Pathogens. 2022 May 5;11(5):544. doi: 10.3390/pathogens11050544 (PMC9146669; doi:10.3390/pathogens11050544)
Supplement: Supplementary file 1 [file pathogens-11-00544-s001.zip › Supplementary Materials.pdf]

pathogens

MDPI

Supplementary Materials

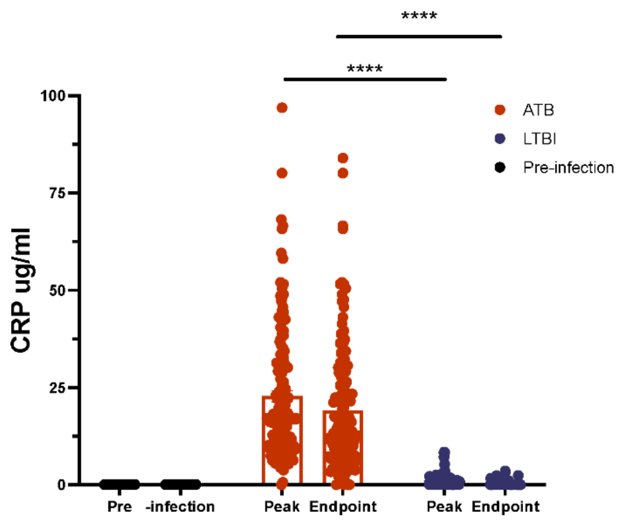

Supplementary Figure S1: CRP values at Pre-Infection, Peak, and Endpoint. CRP values observed during pre-infection, peak, and endpoint varied drastically between ATB (red) and LTBI (blue). (****, P value of <0.0001).

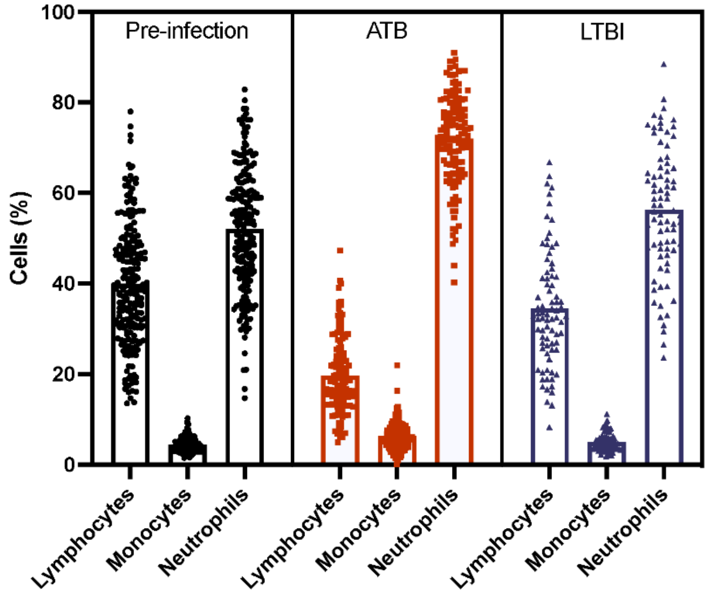

Supplementary Figure S2: Pre-infection and Endpoint Cell Percentages. Pre-infection (black) and Endpoint (red) and LTBI (blue) percentages of cells in peripheral blood.

PBMC

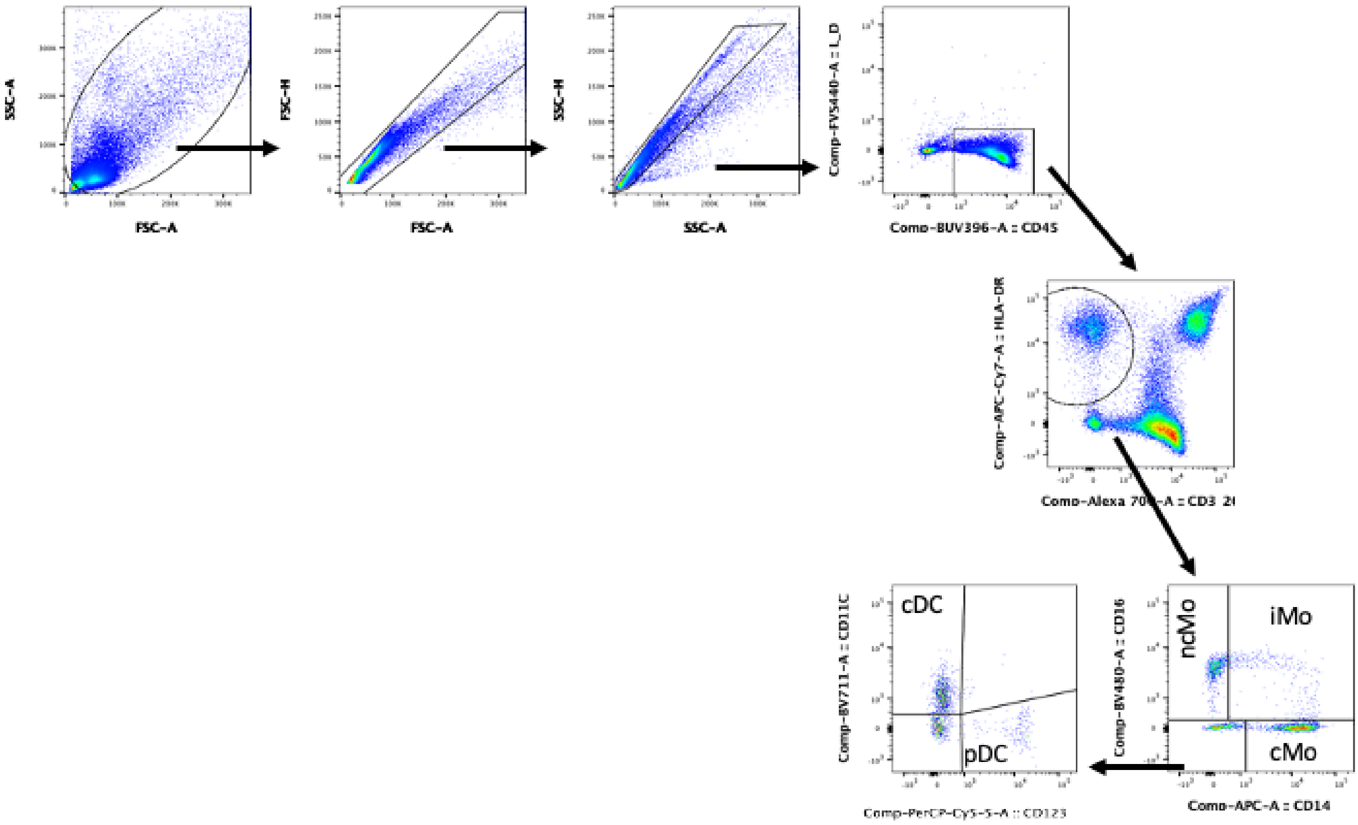

Supplementary Figure S3: Flow cytometry gating strategy for PBMCs.

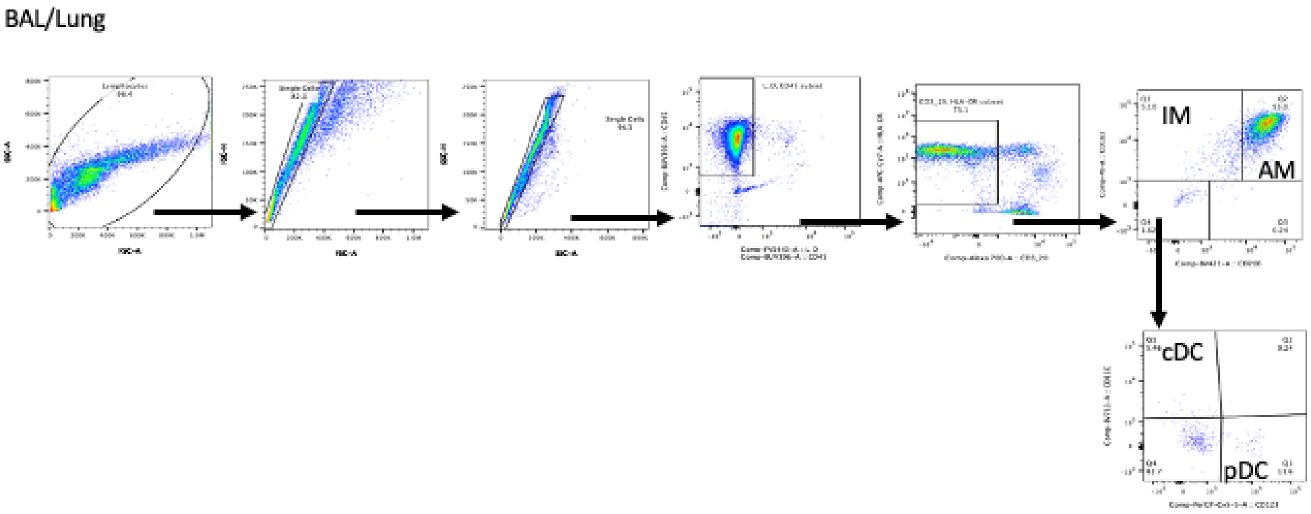

Supplementary Figure S4: Flow cytometry gating strategy for BAL and Lung tissue cells.
